# Autism and bilingualism: A thematic analysis of practitioner perspectives in the United Kingdom

**DOI:** 10.1111/1460-6984.12939

**Published:** 2023-07-30

**Authors:** Rachael Davis, Farah Binti Mohd Zaki, Lesley Sargent

**Affiliations:** ^1^ Division of Psychology, Sociology & Education Queen Margaret University Edinburgh UK; ^2^ Edinburgh Medical School University of Edinburgh Edinburgh UK; ^3^ East Lothian and Midlothian Speech and Language Therapy Department NHS Lothian Edinburgh UK

**Keywords:** autism, bilingualism, identity, practice

## Abstract

**Background:**

At least 25% of autistic children worldwide have the potential to grow up in a bilingual environment. However, many autistic children are being denied opportunities to access additional languages and the cultural, familial and community connections that come with this. There is little evidence identifying the barriers to language learning and access, and no research addressing the perspectives of speech and language therapists (SLTs), who are crucial in supporting parents to make informed choices about bilingualism with their child.

**Aims:**

The aim of this research was to understand the experiences of SLTs working with autistic bilingual children, to understand the main considerations when working with families, and the opportunities and barriers for training, including the sources of information that current practice is based on.

**Methods and Procedures:**

Twelve SLTs from across the United Kingdom were recruited for this study. All participants were experienced in working with autistic bilingual children and their families (*M* = 7 years, range 4–23 years). Semi‐structured interviews were conducted and focused on the experiences of SLTs regarding familial bilingual experiences, the effect of sociocultural factors of practice, and the extent to which practice is based on current research.

**Outcomes and Results:**

Data were analysed using reflexive thematic analysis. Three central themes were identified from the interviews: (1) participants discussed parental uncertainties as to whether they were doing the right thing for their child, (2) while participants were in support of bilingualism, they were not always confident that they were providing the right advice and found it difficult to in keep up to date with relevant, evidence‐based research, and (3) participants highlighted a need to shift towards a more inclusive and culturally diverse practice.

**Conclusions and Implications:**

This is the first qualitative study to understand the perspectives of SLTs working with autistic bilingual children. We identify several key difficulties in supporting access to language learning, and these findings have immediate and longer‐term implications for supporting SLTs, and in turn, the children and families they support.

**WHAT THIS PAPER ADDS:**

**What is already known on the subject**

- Research suggests that autistic children currently have fewer opportunities to maintain bilingualism compared to neurotypical peers. Despite the lack of evidence, many families remain concerned that bilingualism will have a negative impact on their child's development. To date, little is known about the perspectives of speech and language therapists (SLTs) who play a significant role in supporting the development of autistic bilingual children. This is the first study to provide an in‐depth qualitative analysis of the experiences of SLTs working with autistic bilingual children and their families in the United Kingdom.

**What this study adds**

- The results highlight a number of reoccurring barriers in providing optimal support: first, frequently cited concerns about bilingualism from parents that link to a lack of understanding about autism and the role of SLTs more generally. Second, SLTs do not have confidence in the assessments and tools available and described a lack of emphasis on cultural factors in practice. Many SLTs were concerned about the limited options for resources and interventions available in other languages, which could be challenging for parents who were less proficient or confident communicating in English. Third, SLTs reported having limited opportunities to keep up to date with relevant research to support their decision‐making processes.

**What are the clinical implications of this work?**

- These results have several important implications for practice—they highlight the need for more inclusive practices where possible, a need for more diversity within the profession and further opportunities to be provided with evidence‐based advice around good practice. The results also suggest a benefit of providing accessible, evidence‐based resources for parents about autism and bilingualism, to ensure that research key findings are reaching families.

## INTRODUCTION

### Bilingualism and neurotypical development

Bilingualism can be defined as the acquisition and use of two or more languages (Lehtonen et al., 2018). It is estimated that at least 50% of the world's children have the opportunity to grow up in a bilingual environment (Marian & Shook, 2012), with evidence of increasing prevalence in the United Kingdom (Edwards, 2011).

There is a substantive literature focusing on the effects of bilingualism on development in neurotypical children. To date, research overwhelmingly reports no negative effects of bilingualism (Bialystok, 2021; Gunnerud et al., 2020), and possible benefits in some cognitive domains (e.g., Kovács, 2012; Marian & Shook, 2012). Research also highlights the significant impact of bilingual exposure on socio‐emotional factors including well‐being, family bonds and comprehension of moral values, beliefs, identities, norms, ideologies and expectations (Halle et al., 2014; Opitz & Degner, 2012). Bilinguals are shown to express different cultural and social expectations when switching languages (Panayiotou, 2004), and although children can have a good vocabulary in a second language, they do not necessarily process the words with the same emotional connection (Pavlenko, 2005). Research also suggests that parents can perceive a closer emotional bond with their children when speaking in their primary language and report feeling more able to connect emotionally with their child (Opitz & Degner, 2012).

### Bilingualism, autism, and development

Until recently, bilingualism was rarely discussed in relation to people with neurodevelopmental conditions, including autism (Drysdale et al., 2015). Although research is growing in this area, the evidence base is still relatively limited (Davis et al., 2021). However, research findings to date are comparable to those with neurotypical children. Either null effects or tentative positive effects have been reported relating to language development (Beauchamp & MacLeod, 2017), facets of social development (Valicenti‐McDermott et al., 2013) social attention (Davis et al., 2021) and executive functions (Gonzalez‐Barrero & Nadig, 2019; Montgomery et al., 2021; Sharaan et al., 2021). To date, no long‐term negative effects have been found in any domain (for a review see Drysdale et al., 2015; Lund et al., 2017; Wang et al., 2018).

Research has also highlighted the potential benefits of a bilingual environment on socio‐emotional well‐being and family bonds for autistic children^1^ (Halle et al., 2014; Han & Huang, 2010). For example, Kremer‐Sadlik (2005) interviewed immigrant parents with autistic children and found lower levels of family cohesion (e.g., children were less likely to participate in family conversations or parents did not address their child often) and a higher likelihood of communication breakdown between parents and children when parents chose to speak only English with their children. Parents who used their primary language with their autistic child reported feeling closer and more connected—not only because of reduced language barriers, but also knowing that they are sharing their heritage and identity (Kim & Roberti, 2014; Yu, 2013). Hampton et al. (2017) also highlights the link to primary language use and family bonds for parents of autistic children in particular, with primary language use being associated with high levels of affection and emotional expression between parents and children.

Given the prevalence of both autism and multilingualism, and the impact of bilingualism across cognitive development and well‐being, it is crucial to ensure equal access to language learning. However, research suggests that children with neurodevelopmental conditions currently have fewer opportunities to maintain bilingualism compared to neurotypical peers (Pesco et al., 2016). Although some families are positive about the impact of bilingualism, many remain concerned that bilingualism will have a negative impact on their child's development (Hampton et al., 2017; Howard et al., 2021a). Research suggests that practitioners can also share these concerns. Indeed, a number of studies asking parents about the advice they received from practitioners suggests that maintaining a monolingual, majority language environment is often assumed to be the best option for autistic children (Hampton et al., 2017; Jegatheesan, 2011; Kay‐Raining Bird et al., 2012).

Despite these concerns not being supported by evidence, parents worry that there will be detrimental effects on language and cognitive development, or that bilingualism will exacerbate potential language delays (Hampton et al., 2017; Yu, 2013). It is clear from research with families that findings are not always filtering to parents. This could be, in part, due to speech and language therapists (SLTs), educators and clinicians lacking confidence in providing advice for parents and a general lack of accessibility to jargon‐free and evidence‐based guidelines for families. What is less clear, however, is the perspective of speech and language, education, and health professionals, who play a significant role in supporting autistic children and their families (Uljarević et al., 2016).

### Autism, bilingualism and practitioner experiences

Research has predominantly focused on the views of parents (Hampton et al., 2017; Howard et al., 2021b; Yu, 2013) and there is currently little evidence regarding the perspectives of practitioners. In educational settings, Howard et al. (2019) found that while staff generally held positive views of bilingualism, this was not always congruent with practice. Many expressed concerns that language learning would not be feasible for all autistic pupils and could have negative consequences on learning for children with language delays. Furthermore, Pesco et al. (2016) found that autistic children attending school in the majority language do not receive the same opportunities for exposure to their primary language as neurotypical peers. The authors argue for stronger collaborations between schools and SLTs to ensure support for children across settings.

To date, only one survey‐based study has elicited the views of SLTs (Marinova‐Todd et al., 2016). In a survey study, school and clinic‐based practitioners in Canada and Europe were asked about the availability of bilingual language services and attitudes around bilingualism for autistic children. As with educators, respondents demonstrated positive attitudes towards bilingualism and are aware what ‘best practices’ *should* look like. However, the authors report a disconnect between practice and opinion; findings suggest that the needs of minimally verbal autistic bilingual children or autistic children with an intellectual disability in particular are not being adequately met, and that findings from research are not always reflected in practice. This research provided a useful first step into understanding the attitudes around by bilingualism. However, an in‐depth exploration of the specific barriers and opportunities in practice are needed to understand the influence of practice on children and families.

As the number of families raising children in a bilingual environment continues to increase, there is a clear need to address the gaps between research and practice (Wilder et al., 2004) and to conduct further research that addresses the experiences of SLTs working directly with autistic bilingual children and their families. Research highlights inequalities around access to language learning for autistic children, leading to calls for a better understanding of contextual factors that relate to autism and bilingualism (Davis et al., 2021; Howard et al., 2021a; Sher et al., 2022). Crucially, Marinova‐Todd et al. (2016) identifies a need for a greater insight into SLT experiences and perspectives from those who work with autistic bilingual children. No study to date has qualitatively explored the perspectives of SLTs who work with autistic bilingual children. Therefore, the aim of this study is to provide an in‐depth qualitative analysis of the experiences of SLTs in the United Kingdom providing support for autistic bilingual children and their families. Given the disconnect between research and practice, this study was guided by the following research questions:

1. What are the main factors that SLTs consider relating to attitudes and beliefs of families of autistic bilingual children?
2. What are the experiences of SLTs who work with autistic bilingual children?
3. What information do SLTs base their practice on?

## METHODS

The study employed a qualitative design using semi‐structured interviews, that were analysed using thematic analysis (Braun & Clarke, 2006, 2021, 2013). Ethical approval was obtained from the University of Edinburgh Psychology Research Ethics Committee.

### Participants

Participants were 12 SLTs (see Table 1 for demographic information) who fulfilled the following eligibility criteria: (1) all participants had gained experience working with bilingual autistic children and their families in clinical and/or school settings in the United Kingodm for at least 2 years; and (2) SLTs were based in the United Kingdom. Participants were based in Scotland (*n* = 5) and England (*n* = 7). This criterion ensured that participants could draw from recent and relevant experiences providing support to autistic bilingual children. Participants were recruited through SLT networks and targeted online advertisements.

**TABLE 1:** Participant demographics

	Age (years)	Level of education	Gender	Ethnicity	Location	Area of practice	Language status	Years in practice
1	20–29	Postgraduate	Female	White	Scotland	SLT	Bilingual	20
2	20–29	Undergraduate	Female	White	England	SLT	Bilingual	3
3	20–29	Undergraduate	Female	White	England	SLT	Monolingual	6
4	20–29	Postgraduate	Female	Asian or Asian British	England	SLT	Bilingual	4.5
5	20–29	Postgraduate	Female	White	England	SLT	Monolingual	3
6	30–39	Postgraduate	Female	White	England	SLT	Monolingual	5
7	30–39	Undergraduate	Female	White	London	Specialist SLT (autism)	Monolingual	3
8	40–49	Postgraduate	Female	White	Scotland	SLT	Monolingual	6
9	40–49	Postgraduate	Female	White	Scotland	Diagnosis of autism/communication conditions	Bilingual	22
10	40–49	Postgraduate	Female	White	Scotland	Specialist SLT (autism)	Monolingual	14
11	50–59	Postgraduate	Female	White	England	SLT	Monolingual	7
12	60–69	Postgraduate	Female	White	England	SLT	Monolingual	37

*Note*. All names have been replaced with participant numbers to ensure the anonymity of participants. Ages have been put into decade bands to preserve anonymity of participants.

Abbreviation: SLT, speech and language therapist.

### Procedure

All participants provided written informed consent before taking part in the study. Interviews were conducted in English by the second author (FBMZ) in a one‐to‐one setting. Interviews were conducted in person or over the phone depending on participant preference and feasibility. Interviews lasted between 35 and 45 min and were audio‐recorded to allow for transcription. Participants were provided with information about the study and their right to withdraw from the study at any time. They were also told that they could choose not to answer specific questions and go back to previous questions at any point.

### Measures

Data were collected using a semi‐structured interview tool, developed for this study by the research team and in consultation with a stakeholder advisory board. The advisory board were recruited to consult on a larger autism and bilingualism project see Davis et al. (2022). The group included an autistic multilingual adult, a parent of a multilingual autistic child, a bilingualism and language researcher, and an SLT who works with autistic bilingual children and is a co‐author here (LS). Following a review of current literature, there was no suitable pre‐existing interview measure for the current research questions. In order to create the questions, we presented the literature review to the advisory board at an in‐person meeting. As a group, we suggested and redrafted an initial set of questions, each of which was discussed and agreed upon. Advisory members had an additional week to send any feedback and questions were finalised via email. The final set of interview questions consisted of 10 open‐ended questions and was used as a guide during the interviews. See Appendix B for the interview questions. This semi‐structured approach allowed for interviews to be more focused on exploring the points raised by participants’ responses and provided flexibility in allowing for follow‐up questions (Barriball & White, 1994; Kallio et al., 2016).

### Data analysis

All interview data were analysed following a reflexive thematic analysis (Braun & Clarke, 2013, 2019), and we adopted a critical realist approach, following the assumptions set out by Maxwell (2012). Maxwell argues that while a real world exists independently of beliefs and theories, our subjective understanding of this world is framed by our own specific experiences and world lens. Therefore, rather than looking for an objective truth through qualitative analyses, we were open to all interpretations and perspectives of reality.

Reflexive thematic analysis can be defined as a method for identifying, analysing, and reporting themes or patterns within the data (Braun & Clarke, 2013). The main advantage of this approach is the flexibility in terms of coding—being able to derive semantic and latent meanings from the data set and allowing for a combination of deductive, theory driven, and inductive, data driven approaches. Our research questions indicated the overarching areas we wanted to explore, and our themes were deductively guided by the research questions. However, we inductively identified subthemes and used open coding to ensure that first level codes best represented the meanings of participants (Braun & Clark, 2013).

In line with Braun and Clarke (2013, 2019), the six‐phase framework of analysis was employed to identify key themes in the data, including familiarisation, coding, theme development and review. RD and FBMZ read and re‐read the transcripts, and individually identified areas of interest by labelling data with key words or ‘codes’ such as ‘strategies, not rules’ and ‘parental preference’ from meaningful repeated patterns. Codes were discussed and revised based on the meanings they had identified in the data, and repetitions and similar codes were eliminated in the reviewing process to prevent overlapping and ensure internal coherency. FBMZ and RD grouped codes to form subthemes and overarching themes. Using an iterative process, FBMZ and RD discussed and reviewed each theme and subtheme, focusing on internal coherence and separating or combining themes where necessary. Themes and subthemes were reviewed and checked by RD to ensure they related to the original coded data.

Positionality is an important consideration in qualitative research. In terms of positionality, all authors identify as non‐autistic. All authors follow neurodiversity‐based approaches to understanding autism and reject a medicalised model. One researcher is an SLT (LS). We used group discussion to consider ways in which the authors’ prior knowledge and experiences could influence any analyses. Two researchers are bilingual (FBMZ, LS) and one researcher is monolingual (RD).

## RESULTS

**FIGURE 1:**
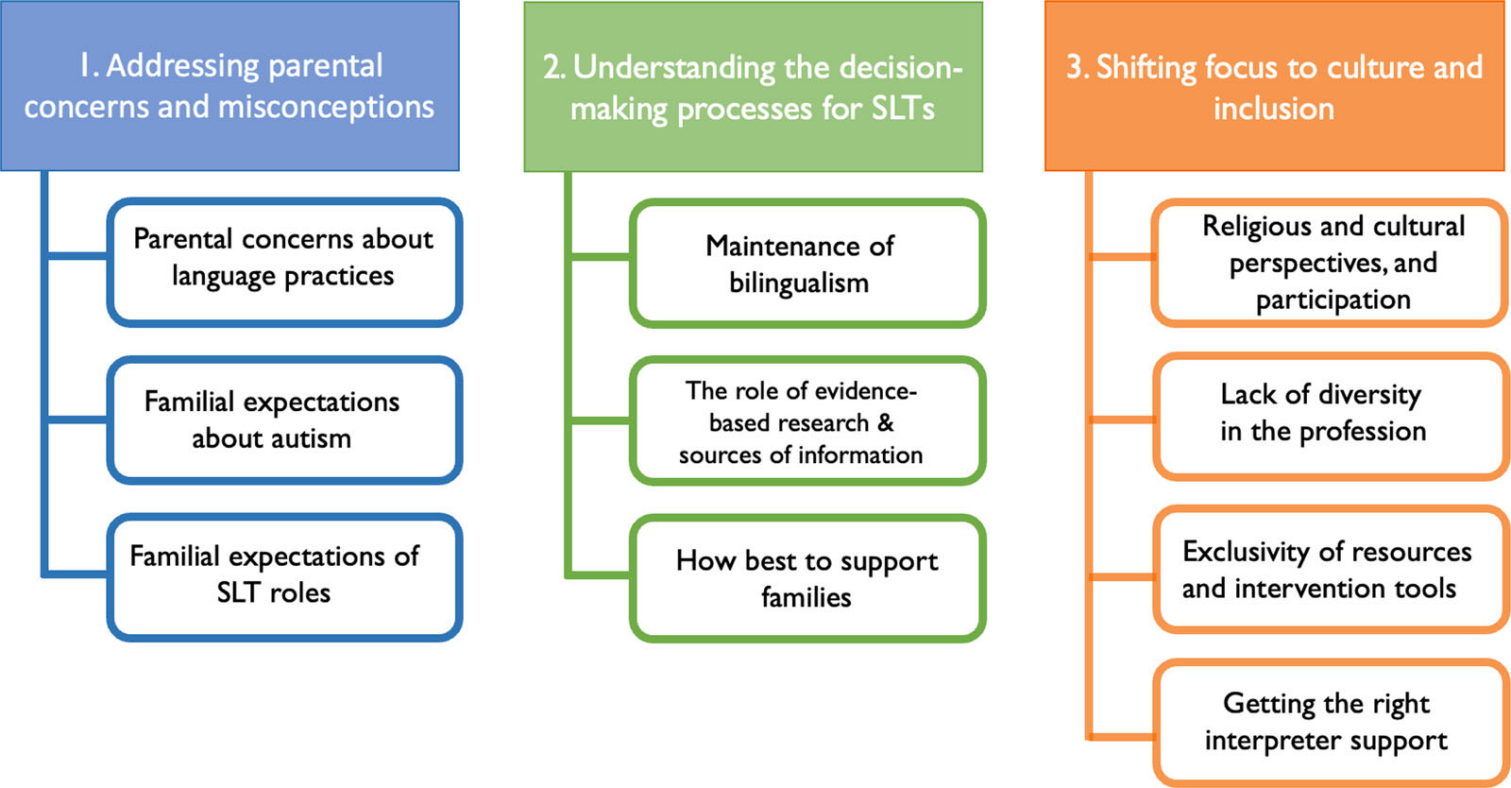
Structure of themes and subthemes. Abbreviation: SLT, speech and language therapist. [Colour figure can be viewed at wileyonlinelibrary.com]

In the following three themes, we explored SLT perspectives around working with bilingual children and their families, with a focus on specific considerations regarding practice and barriers and opportunities for training and support. As seen in Figure 1, these themes relate to (1) professionals’ experience with parents, and parental concerns over whether they are doing the right thing for their child; (2) understanding the decision‐making processes of SLTs working with autistic bilingual children; and (3) the need to shift toward a more inclusive and culturally diverse practice. Quotes are provided in the text and participant names have been anonymised and replaced by participant numbers.

## THEME 1: ADDRESSING PARENTAL CONCERNS AND MISCONCEPTIONS

### Subtheme 1: Parental concerns about language practices

SLTs identified several parental concerns that arose most frequently during consultations with parents. First, whether English should be the only language spoken at home with their child, and whether growing up in a bilingual household could cause additional delays, confusion or prevent their child from developing language:

They use their home language but then when they realise that the child's got language difficulties, they think oh should I just speak English to them, are they going to be confused with the two languages. (SLT 11)

That's probably one of the main concerns – whether or not they should be speaking English. Along with obviously, the main concerns like when will my child talk and what will the future look like? (SLT 7).

This was also the case for parents who speak different languages from one another: ‘If one parent speaks in one language and the other speaks in another, they ask, “should we both be speaking the same language to the child?”’ (SLT 2). SLTs also reported that parents who were less proficient or confident speaking English were often concerned about the impact they may have on their child's learning. For example, parents were worried about passing on incorrect grammar or vocabulary:

One parent particularly reported that she's concerned she can't give her son a good enough English model because her English is limited. So, she worries about his grammatical formation because hers isn't a typical English pattern, and he won't answer her in her home language, so she started using English with him, but she's concerned that she's impacting him on his language development. (SLT 7)

What if they say something that is not correct in English, or they make mistakes and the child then learn the incorrect form. So, there is all this fear around “oh do I use English? Do I use my home language?” (SLT 10)

### Subtheme 2: Familial expectations about autism

SLTs felt that some parents’ beliefs about autism are framed by what they know from their home context outside of the United Kingdom:

If you've only ever been in a society where the people who are diagnosed with autism have complex needs and significant learning difficulties, then you might struggle to recognise that in your child that is actually highly able but has a subtle communication difficulty. (SLT 4)

Some parents might feel that because you've good language, it means that you can't have ASD [autism spectrum disorder] (SLT 9).

SLTs felt this also affected choices later in the child's life, such as schooling decisions, when many families arrive in the United Kingdom from countries where views of autism might be considered stigmatising:

There's perhaps also an assumption based on what would happen in their home country, so I think sometimes we have families who'll be reluctant to acknowledge to us that their child might have ASD because they're anxious that that might result in their child not being able to go to a mainstream school or being held back or being stigmatised in some way. (SLT 1)

Some families are very comfortable with sending their child into a special needs school, some of the parents would not. So that would be really hard for some to consider that their child might not be able to go to a mainstream school. (SLT 2)

SLTs expressed that in the context of bilingual families, many parents were unaware of the social and communicative differences often associated with autism and have attributed delays and/or difficulties to growing up in a bilingual household, rather than a developmental condition.

‘In the context of families who are bilingual, it can be even more complicated, because they might attribute the child's difficulties to the fact that they're learning more than one language, so they might kind of feel, “well you wouldn't expect my child to be socialising in nursery because actually, the language at nursery is different from the language that the child is most comfortable in at home”’. (SLT 4)

### Subtheme 3: Familial expectations around the role of SLTs

A number of participants reported a divergence between the expectations of family members and SLTs themselves regarding their role, which are often based on knowledge and experiences from the family's home country. For example, many SLTs stated that their role is frequently regarded (by parents) to be the driving force behind positive changes and outcomes, yet this is not aligned with the views of SLTs themselves:

They believe that we [professionals] are the only people who are able to provide the specialist help that the child needs, whereas…we really believe that parents can make the biggest difference. (SLT 11)

Sometimes their expectation is that the medical professional is going to do a test and is going to give therapy and that therapy will resolve the problem. (SLT 8)

Some SLTs had experienced families’ preferences to turn to alternative methods in efforts to ‘cure’ their child. These families were often less aware of the autism and role of SLTs, and this was notable in their interactions with parents: ‘due to cultural differences, *some families don't trust professionals much… some families say, “I'm going to take my child to a healer”’* (SLT 6).

## THEME 2: UNDERSTANDING THE DECISION‐MAKING PROCESSES FOR SLTS

### Subtheme 1: Maintenance of bilingualism

SLTs overwhelmingly expressed their support for maintaining a bilingual environment and that they would generally never advise against autistic children using or maintaining multiple languages:

I wouldn't, I would never advise against it [bilingualism]. (SLT 11)

I feel like, if I were a bilingual parent and somebody were to tell me not to speak in my mother language to my child, that would be really hard for me to hear. (SLT 1)

I would encourage the families to use whatever they feel comfortable with, so if they feel comfortable at home talking in their native language and communicating in their native language, I would encourage them to do so and I would do my best to support them in that sense. (SLT 6)

SLTs did mention exceptions to promoting a bilingual environment that were related to family preferences. For example, if parents decided to bring in the other language much later in the child's life, some SLTs thought that it might arguably be ‘too late’: ‘*Potentially, if it was a thing where parents wanted to introduce it much later in life, if the child is 10 years old and they decided to… But I wouldn't necessarily advise against it’* (SLT 4).

One SLT felt that the child's cognitive ability may potentially be a factor to consider a monolingual environment: ‘If you've got a child who's very, very cognitively impaired, or very language impaired, and they've got very little language and they're not making any progress…. that might be one strategy to take for a while’ (SLT 9).

Most notably, SLTs who actively supported maintaining bilingual households described three central reasons that they relay to parents who are concerned. A number of participants discussed how important a good model of language was for children, and that often is optimal when parents speak in their home language: ‘*what we want the children to hear is a model of language that is natural, that has the natural intonation, pronunciation and grammar and breadth of vocabulary and you're only going to get that if you're speaking in your mother tongue* (SLT 1).

Second, many also highlighted the importance of maintaining the bilingual environment for the child to preserve cultural values and relationships in the family:

I think it's quite culturally important as well… if they have family members who only speak that other language, then it's important for them to be able to communicate with the child. (SLT 4)

I have parents from two different cultures, although they both speak English, there's definitely some connections with some parts of their background… I worry sometimes that if the children lose that. (SLT 3)

Third, two SLTs mentioned a support for maintaining bilingualism because they have read studies describing the potential cognitive benefits:

There have been many studies to show that bilingualism is great for the brain development. (SLT 2)

There's neurological benefits and children are shown to do well and have better kind of executive function… about bilingualism in general. (SLT 3)

### Subtheme 2: The role of evidence‐based research and sources of information

Despite their common support for bilingualism, the majority of SLTs shared uncertainties as to whether they are providing the right advice to families associated with a lack of evidence‐based information in the area:

I'm advising parents to use both languages but actually I don't know if there's sort of a strong evidence to support either way. (SLT 7)

To be honest with you, I feel like it's something that I personally could get a wee bit more understanding of ‐ I feel like it is an area where it is a real lack of understanding. (SLT 1)

When asked about the main sources of information that were relied on for their practice, SLTs commonly cited guidelines provided by the Royal College of Speech and Language Therapists (RCSLT):

First of all, it's stated in our Royal College of Practitioners, the guidelines around that, it says quite overtly that it's not something that can hinder the acquisition of language. (SLT 4)

We follow the RCSLT guidelines quite closely, so it's really important to keep up to date with those. (SLT 1)

However, the majority of SLTs identified a lack of dedicated time for wider reading as a potential barrier for understanding specific subject areas, including autism and bilingualism. Many felt that there were fewer opportunities than they would like to keep up to date with new research findings due to their busy schedules:

I have to be honest, I'm not often, just because of the pressures of practice and caseloads, there's not a lot of time to put aside for some more specific research. (SLT 7)

I don't always know what to advise if I'm completely honest. I suppose I'm going by case‐by‐case basis rather than having a good, solid understanding of what research would say, what the evidence base is. If you could guide me towards any, that would be great. (SLT 3)

### Subtheme 3: How to support families

As a group, the theme of support regarding children and parents was clear from all SLTs. Most notably, they emphasised the aspects of their role relating to ‘supporting’ the parents they work with, rather than making important decisions for them:

I don't think my role is saying to a family, “you should definitely do this” ‐ our role is more in supporting the child's communication skill rather than telling the family what to do (SLT 6).

We really believe that parents know their children best, they are with them the most and so they are the people who can make the biggest difference. It's our job really, to try and kind of help them to understand the best ways to do that. (SLT 8)

Additionally, SLTs agreed that when working with children, a priority is to provide a holistic and supportive role, to promote participation and inclusion in society, rather than a focus on ‘treating’ autism: *‘it's important that we are helping these children to be active and happy in their world and thinking about bilingual and autism as a part of that’* (SLT 5).

## THEME 3: SHIFTING FOCUS TO CULTURE AND INCLUSION

### Subtheme 1: Religious and cultural perspectives and participation

Participants described the importance of considering cultural and religious factors when meeting families. For example, many highlighted the importance of gaining a wider appreciation of cultural viewpoints, and the subsequent impact of this on expectations, instead of placing their own expectations on those families: ‘*Being aware that whatever spiritual or cultural support gives is really important and can have a big impact on their attitude and ability to engage with therapy*’ (SLT 3). Considerations of cultural and religious factors also included more practical aspects: ‘*I wouldn't be offering appointments at a time that I know somebody would be worshipping, from a religious point of view*’ (SLT 7).

SLTs emphasise the importance of having some consideration or understanding of the language environment the child is in, and how this links to their cultural background when interacting with bilingual children:

We had an autistic boy who only made certain sounds… we just thought it was a sound that he made, if we said it back to him he would get really excited and really enjoyed it and then when we had a staff member who spoke the same language as his parents join our team, she told us that in that language, that actually meant something, like ‘my love’ or something. So, it was really meaningful. (SLT 4)

Conversely, one SLT expressed that while cultural understanding was important, assumptions should not be made about clients they interact with, solely based on the culture they belong to: ‘*I'm not the kind of person who says ‘ok, so I'm seeing somebody from Somalia, I better look up what Somalis are like’, because I don't really believe in that ‘cookbook’ approach in how to communicate culturally*’ (SLT 12).

There were mixed responses to the impact that this understanding of cultural and religious factors had on local community participation for families. In this sample, SLTs working in urban areas (and in areas with overall greater cultural diversity) felt less concerned about children not being able to participate in community‐based activities compared to less diverse areas.

Here, there's a lot of emphasis on cultural awareness in school, and celebrations kind of, appreciating religious festivals and there's a lot of staff from the local community. (SLT 5)

I think, because we live in [a city], there's so many different nationalities. I think nowadays its very common to see a child with ASD who is also bilingual and to be honest, I think where we have a role, is just to support them and to encourage them participate as bilingual children, and not to dismiss the fact that they are bilingual. (SLT 11)

Conversely, SLTs working in less diverse areas report that some bilingual parents and families who are new to the United Kingdom often feel ‘socially isolated’, and ‘*might not be able to participate in parent‐to‐parent learning or social activities within existing parent groups*’ (SLT 8):

They [parents] often don't have adequate English to come to these groups, they're not meeting other families and I think sometimes there is a sense of isolation for the parents where they're bilingual but not to the extent where they can join with other groups. (SLT 4)

It would be nice to put parents in touch with each other. I think that that has a whole raft of confidentiality attached to it. But I do think that isolation can be difficult particularly because their children can be so complex ‐ we see a lot of parents sharing information with each other and it's so valuable. (SLT 7)

### Subtheme 2: Lack of diversity within the profession

While acknowledging the importance of embracing cultural and religious beliefs, SLTs identified reoccurring barriers to optimally support some parents and children. First, participants discussed a lack of cultural diversity within the profession, with suggestions that this lens could be slowing progress within the profession:

We do our best to know what cultural factors are, but I don't think we always get it right. I think we probably make assumptions as we are a very female, very middle class, very Western profession ‐ practitioners on the whole. (SLT 8)

The diversity of SLT's is a massive issue. We're a team of 6 SLTs working with probably 75–80% [of clients with] English not as a first language. We are all from the UK, we're all white, we're all women and I think that is the case for also a lot of other places and I think that needs to be improved urgently. I think that's something we need to like, address through the way we are attracting SLTs. (SLT 5)

### Subtheme 3: Exclusivity of resources and intervention tools

Participants highlighted a number of barriers in providing support to some children and families, primarily because of the tools available to them. For example, concerns were raised about the limitations of current resources and parent training programmes. Some of the materials and resources available for use in sessions families were often felt to be culturally inappropriate, for example: ‘*There are for some groups for example, that it would be inappropriate to use pigs—but we have quite a lot of resources and pictures of pigs and games that involve pigs and sometimes we realise that a bit too late and we have had to apologise to the family*’ (SLT 8).

Others shared that differences in parent–child interaction styles could make some intervention and support‐based practices more difficult: ‘*child rearing styles are kind of different, or it might be quite inappropriate to sit on the floor, for example*’ (SLT 10).

In regard to diagnostic assessments, participants raised concerns that current tools are not always inclusive or relevant for all children. SLTs described difficulties in differentiating behaviours commonly associated with autism in a Western context and potential differences in cultural norms. An example that was raised was the use of eye ‐contact. One SLT explained that in some cultures, children are expected to not make eye contact when interacting with adults; this SLT had conducted a research project in Bangladesh to observe adults and child interactions where the cultural norm was for children to look away from adults when they were being spoken to: ‘*This for me, was like a ‘blinding light’. I had seen behaviours in Bangladesh, which I had also seen in* [UK city], *and which might unfortunately have been construed as “this child does not know how to make eye contact’*” (SLT 12).

Professionals were also concerned at the limited options for resources and interventions available in other languages, which could be challenging for parents who were less proficient or confident communicating in English. ‘*I also feel that it's difficult that we can't always offer an equity of service because a lot of our programs are delivered in English* (SLT 7)*’*. This issue was thought to be ‘*difficult to overcome realistically as it is heavily related to funding, regulations, and the number of bilingual professionals available’* (SLT 9).

One SLT who worked mostly with non‐speaking children in a culturally diverse area reported a relatively more positively towards this topic: ‘*A lot of staff are also from Bangladesh, so they also speak the home language of a lot of our children, so they are available to support if anything comes up*’ (SLT 10). Other SLT's felt that recognising and developing an understanding and appreciation of different cultures is important and this could be achieved by speaking to the families and maintaining a good relationship with them:

In some cultures, there's less direct interaction with their children… and that might change the decisions I make about the sort of interventions that you might choose. (SLT 9)

It's just knowing the family and being able to have those open lines of discussions just to ask them about their feelings towards these things. (SLT 4)

### Subtheme 4: Getting the right interpreter support

The use of interpreters emerged as one of the main considerations for SLTs working with bilingual families. SLTs reported that working with an interpreter in the room can be a positive and useful experience for families: ‘*Having an interpreter in the room who speaks their language it can be quite reassuring, that they've got somebody else there*’ (SLT 1).

However, SLTs expressed overlapping concerns relating to the use of interpreters. Difficulties in finding appropriate interpreters was cited as additional challenge, and there was a high level of variability in terms of the language required and diversity of populations in the area in which they practiced: ‘*there's a big Polish community, so it's quite easy to get a Polish interpreter. Other languages, its actually quite tricky to get one. I think I had somebody who needed a Tamil interpreter, and I couldn't get one… They were going to provide me with a telephone interpretation service, and I thought that's going to be really difficult*’ (SLT 11).

At times when interpreters weren't available, SLTs noted that additional family members frequently offered support as an interpreter. This situation was thought to have both advantages and disadvantages; where on one hand the family might be more comfortable, on the other hand, parents can be overly involved in the process and can find it more difficult to remain neutral. For example:

They would want to show their child off in the best possible light, or even go the other way and say ‘oh they can't do any of it’ when actually they might be able to do a little bit. (SLT 2)

What I'm looking for is subtle things about the way they use their language, and a parent…they'll give an interpretation of what the child is saying but what I want to know is exactly what the child has said, like word for word. (SLT 9)

Some described occasions where they were not confident that information was always being accurately conveyed to families, and that translators sometimes appeared to add their personal input rather than exact translations from SLTs:

I don't know what the interpreter is saying, and sometimes interpreters do seem to talk for 20 min and then when you say to them “what did you say?”, they say “oh I just said to the mother that it would be a good idea to play with the bricks”. Or sometimes interpreters will say “I've just given them some ideas and suggestions”. (SLT 8)

Sometimes there's a bit of a mismatch with the information that we're giving and providing to support the interpreter and what they're actually getting. I might put that forward on my referral form, but the interpreter just gets the name, the date, the place, they don't get all that background information they need. (SLT 7)

In a similar vein, some participants also reported difficulties knowing whether interpreters have followed their instructions correctly which can be problematic, particularly during standardised assessments. For example, diagnostic assessments for autism typically require the use of exact language or for SLTs to use specific verbal instructions with the child. SLTs raised concerns that imprecise translations or repetition of instructions could reduce the validity of such assessments:

During an assessment they [interpreters] need to say things exactly how you say it and they can't repeat. Because obviously a lot of our assessments here you're only allowed to repeat it once, so you're not allowed to repeat it. So, there are little rules that interpreters might not know, so there can sometimes be implications there that you need to consider. (SLT 1)

You have no means of telling whether the interpreter has interpreted exactly what you want. And all the kind of little nuances, you're not sure if they've got those. We do assessments with children, and I give quite specific instructions to the interpreter, I say “I want you to say this but please don't say that”. (SLT 11)

## DISCUSSION

This study sought to better understand the perspectives and experiences of SLTs working with autistic bilingual children and their families. This study was undertaken to fill the knowledge gaps where previous research has only reported qualitative findings relating to professional practice through parent perspectives (e.g., Yu, 2013), by directly asking SLTs themselves. Overall, this study identified three key insights into SLT practice: (1) SLTs frequently experienced anxiety directly from parents, who questioned whether they were making the best decisions for their children's development—though in part this was due to parent misconceptions about autism and their views on the role of SLTs. (2) Difficulties supporting families were partly due to a lack of diversity in the field and a lack of linguistically and culturally relevant tools and resourcing to support their practice. (3) SLTs felt uncertain as to the scientific underpinnings of the advice they give to parents, citing a lack of time, and accessible evidence‐based resources to direct their practice.

**(1)** SLTs said that many parents still feel uncertain about what the ‘right’ linguistic environment should be for their child and are often worried that hearing more than one language at home will have detrimental effects on the child's cognitive or language development. Furthermore, some parents told SLTs that their child would benefit from hearing English even if they are not confident speaking or communicating in English themselves. This is congruent with previous research with caregivers of autistic bilingual children (Hampton et al., 2017; Kay‐Raining Bird et al., 2012; Yu, 2013). Conversely, research to date has found that prioritising English over speaking a language fluently could result in fewer opportunities for autistic bilingual children to develop both languages compared to non‐autistic children (Marinova‐Todd et al., 2016) and reduced communication between parents and children overall (Kay‐Raining Bird et al., 2012). Given the effects this can have on family bonds (Yu, 2013), it is crucial that we find approaches to relay key findings from research to families.

The current findings also suggest two reasons that contribute to parental uncertainties regarding their child's language environment. First, SLTs discuss the fact that some families have pervasive and stigmatising views about autism that stem from a lack of understanding about what autism is within some communities—for example, believing that autism can and should be ‘cured’ or viewing autism as a narrow and homogenous presentation (e.g., high needs and non‐verbal). This in line with other research findings suggesting that autism is misunderstood, and that autistic people can face particularly stigmatising views in some communities (Hussein et al., 2019; Kinnear et al., 2016; Munroe et al., 2016; Papoudi et al., 2021; Selman et al., 2018) that could lead to further misunderstandings.

Second, SLTs described parental misunderstandings around the role of SLTs—specifically that their role is more akin to a doctor who could ‘treat’ their child. In some cases, this can lead to a lack of trust of professionals and a preference of alternative medicines and therapies. One reason for this could be that perceived social stigma is elevated in cultural minorities across the United Kingdom, and particularly in terms of accessing healthcare (Kandeh et al., 2020; Szczepura, 2005). Therefore, a distrust of clinical professionals for some families is not surprising. Taken together, these findings suggest that a lack of access to research combined with stigma around healthcare and autism in some communities can contribute to parental concerns and fewer opportunities for autistic bilingual children to access multiple languages.

(2) In terms of advice, there was an emphasis on providing support not instructions, and treating every case individually. In this sample, there was overwhelming support for the maintenance of bilingual environments for autistic children regardless of their language or cognitive abilities, and prioritising informed parent choice. Previous research found considerable gaps between research and practice and an uncertainty as to what to advise parents (Kay‐Raining Bird et al., 2012). This discrepancy could be due to the sample, but equally, it could reflect changes in practice due to the recent increase in research in the field, and the subsequent RCSLT guidelines (Pert & Bradley, 2018) provided for SLTs.

Despite this positive outlook regarding bilingualism from SLTs in this sample, many still felt unsure as to the reasoning behind their choice to support the maintenance of bilingualism and were unable to offer families research‐based guidelines. Related to this uncertainty was the barrier of time to research and lack of accessible evidence for SLTs to guide them. Taken together, it is clear that SLTs are making decisions that are supported by evidence but would like to feel more confident in their decision making by having access to ongoing evidence‐based training and resources.

In this sample of participants, while parents were reported to have frequent concerns about the impact of bilingualism, SLTs themselves overwhelmingly supported the idea that autistic children should not be denied access to a bilingual environment. SLTs indirectly discussed some strategies to attempt to alleviate parental concerns. SLTs emphasised to parents the importance of speaking their primary language with their child, and explained how this was crucial for parents who were not confident speaking English. SLTs also focused on the goal of ensuring participation within society and the communities that children are a part of, rather than the idea of treatments. However, as mentioned previously, one of the primary difficulties for SLTs was being unable to question the views of some parents about autism, and the cultural stigma attached to this label.

(3) While SLTs agreed on the importance of considering religious and cultural factors when working with families, they highlighted several barriers to practice. In addition to the lack of diversity within the field, SLTs did not feel that the tools available to them were either culturally appropriate for all children and did not always capture the cultural variability within diagnostic assessments for example. This uncertainty can lead to a lack of confidence, both in terms of providing optimal advice, but also around the validity of using some standardised assessments in clinical settings. This is supported by previous research that tools and strategies used with bilingual children are often culturally inappropriate, particularly when asking families to interact with inappropriate toys or situations that would not by typical in their culture (Mdlalo et al., 2019; Zhang et al., 2006). Relating to autism specifically, this can also lead to misdiagnosis because cultural differences are not accounted for (Harris et al., 2014). Moreover, SLTs were concerned about the equity of service, often having to rely on family members as SLTs, and services needing to be provided in the majority language. Indeed, previous research suggests that this could contribute to parents feeling that they need to speak English in order to actively participate in these provisions (Kay‐Raining Bird et al., 2012).

SLTs also discussed the lack of diversity within the practice. Findings point to concerns over the homogeneity of staff viewpoints, and that this could lead to Western assumptions being prioritised. This lack of diversity is not unique to this sample, and the low representation of ethnic minority SLTs is well documented (Greenwood et al., 2006; Sheridan, 1999).

### Strengths and limitations

This research is the first qualitative examination of SLT experiences working with autistic bilingual children and their families in the United Kingdom. While to date, one survey study has been conducted with Canadian and European SLTs, the current study aimed to explore a broader set of questions around practice in a more systematic way. However, there are some limitations to this study. First, this study included 12 participants who were all based in England and Scotland. Future research should consider widening participation to all countries within the United Kingdom.

Second, the participants in this sample were overwhelmingly in favour of a bilingual environment for autistic children, a view which is not reflected in previous research (e.g., Kay‐Raining Bird et al., 2012). It is possible that there has been a shift in views favouring bilingual environments since he publication of prior papers. However, it is also likely that our recruitment process had an impact on the participants recruited. For example, we utilised known school networks and social media and it's likely that those who took part had a prior interest in bilingualism and autism. One third of the participants were also bilingual, which could also contribute to the high levels of support of bilingualism overall.

Third, all participants identified as female and all but one participant identified as white. While this is somewhat congruent with research findings that white women are generally overrepresented in speech and language therapy (Sheridan, 1999), it is important to note that the experiences here may not be the representative of minority ethnic community members, and more diverse experiences of navigating this area of the profession are required in the future.

## CONCLUSION

It is clear that there are complex issues relating to cultural differences and how best to support families who may have misunderstandings about autism, bilingualism and the role of SLTs more generally. The results suggest that while SLTs are supportive of promoting a bilingual environment for autistic children, parents themselves are still unaware of the potential sociocultural and cognitive benefits of bilingualism. It is clear that providing translated information explaining what autism is, and how autism and bilingualism interact, could be beneficial in reducing stigma and misunderstanding for some families and communities. This is achievable in the short term and could begin help parents to feel more confident in making decisions about their child's cultural and linguistic environment.

This study also exposes a number of longer‐term barriers and areas for change relating to practice. First, there is a clear need for more diversity in the field. Second, it is crucial that SLTs are provided with a wider range of resources and tools to better suit the variability between families from culturally and linguistically diverse backgrounds. Third, it would also be beneficial to offer ongoing access to cultural training and easily accessible, updated research findings. While some of the solutions mentioned above are easier to implement in the immediate future, we hope that identifying these areas for systemic change can in time, lead to greater equity and participation for autistic bilingual children and their families.

## AUTHOR CONTRIBUTIONS

Rachael Davis: Conceptualisation, investigation, formal analysis, original writing. Farah Binti Mohd Zaki: Conceptualisation, investigation, analysis, original writing. Lesley Sargent, Conceptualisation, review and editing.

## CONFLICT OF INTEREST STATEMENT

The authors declare no conflicts of interest.

## Data Availability

Data is available upon request.

## ENDNOTE

1 There is debate regarding the language used when describing autism. Here we use identity‐first language (e.g., ‘autistic person’) as opposed to person‐first language (e.g., ‘person with autism’) to respect the preference of the majority of autistic people (see Botha et al., 2021; Kenny et al., 2016).

## APPENDIX A Final themes, subthemes and example codes

**Table:**

Theme	Subtheme	Example codes
1. Addressing parental concerns and misconceptions	1.1. Parental concerns about language practices	Causing confusion
		Will they catch up?
		Talking possible?
		The future
		Language choices
	1.2 Familial expectations about autism	Denial of autism
		Stigma
		Autism is unfamiliar
		Lack of awareness
		Confusing bilingual and autistic language differences
		Anxiety around difference
		Dated views of autism
	1.3 Familial expectations of SLT roles	Medicalised assumptions
		SLT unknown role
		Finding cure
		Alternative medicine
2. Understanding the decision‐making progress for SLTs	2.1 Maintenance of bilingualism	Never against bilingualism
		Case by case basis
		Parental preference
		Natural model
		Home language is best
	2.2 The role of evidence‐based research and sources of information	Royal college
		SLT guidelines
		No time for research
		Evidence‐based practice
		Accessible resources needed
		Uncertainties on right advice
	2.3 How best to support families	Parent choice
		Autonomy
		Strategies, not rules
		Flexibility
		Communication skills
		Supportive role
3. Shifting focus to culture and inclusion	3.1 Religious and cultural perspectives, and participation	Spiritual/ cultural considerations
		Don't make assumptions
		Practical aspects (timing)
		Remove expectations
		New to United Kingdom
		Social isolation
		Lack of links to other families
	3.2 Lack of diversity in the profession	White
		Western
		Non‐diverse group
		Mostly women
	3.3 Exclusivity of resources and intervention tools	English only
		Understanding barrier
		Inappropriate tools
		Culturally irrelevant
		Westernised
		Lack of non‐English speakers
		Western norms
	3.4 Getting the right interpreter support	Helpful/beneficial
		Risk of misinterpreting
		Technical language
		Logistical difficulties
		Imprecise
		Family interpreter biases

Abbreviation: SLT, speech and language therapist.

## APPENDIX B Interview questions guide

1. Do you have autistic, bilingual children on your caseload? What are they like?
2. What sort of discussions do you have with parents around autism and bilingualism?
3. What is your view as an SLT on bilingualism for children with autism?
4. What would you advise for bilingual parents regarding using more than one language for their autistic children? Why do you feel that way?
5. What are the main factors you consider when advising bilingual parents of autistic children?
6. In what situations would you recommend using/maintaining bilingualism with autistic children? (if any)
7. In what situations would you advise against (if any)
8. What types of information influence your decision or advice to parents?
9. Do you consider the families culture (*if not mentioned in previous answer*) while advising parents?
10. “I'm worried that my child might lose his chance to learn and be part of the culture as autism becomes our priority.” What do you think about this?
11. Is there anything else you would like to talk about or go back to?

Abbreviation: SLT, speech and language therapist.

## References

[jlcd12939-bib-0001] Barriball, K. & White, A. (1994) Collecting data using a semi‐structured interview: a discussion paper. Journal of Advanced Nursing, 19, 328–335. Retrieved May 2019. 8188965 10.1111/j.1365-2648.1994.tb01088.x

[jlcd12939-bib-0002] Beauchamp, M.L. & MacLeod, A.A. (2017) Bilingualism in children with autism spectrum disorder: making evidence‐based recommendations. Canadian Psychology, 58(3), 250.

[jlcd12939-bib-0003] Botha, M. , Hanlon, J. & Williams, G.L. (2023) Does language matter? Identity‐first versus person‐first language use in autism research: a response to Vivanti. Journal of Autism and Developmental Disorders, 53, 870–878. 10.1007/s10803-020-04858-w 33474662 PMC7817071

[jlcd12939-bib-0004] Braun, V. & Clarke, V. (2021) Thematic anlaysis: a practical guide. UK: Sage.

[jlcd12939-bib-0005] Braun, V. & Clarke, V. (2013) Successful qualitative research: a practical guide for beginners. UK: Sage.

[jlcd12939-bib-0057] Braun, V. , & Clarke, V. (2019). Reflecting on reflexive thematic analysis. Qualitative research in sport, exercise and health, 11(4), 589–597.

[jlcd12939-bib-0058] Braun, V. , & Clarke, V. (2006). Using thematic analysis in psychology. Qualitative research in psychology, 3(2), 77–101.

[jlcd12939-bib-0006] Bialystok, E. (2021) Cognitive effects of bilingualism: an evolving perspective. Bilingualism Across the Lifespan, 9–28.

[jlcd12939-bib-0007] Davis, R. , Fletcher‐Watson, S. & Digard, B.G. (2021) Autistic people's access to bilingualism and additional language learning: identifying the barriers and facilitators for equal opportunities. Frontiers in Psychology, 12, 741182. 10.3389/fpsyg.2021.741182 34630254 PMC8492951

[jlcd12939-bib-0008] Davis, R. , Montgomery, L. , Rabagliati, H. , Sorace, A. & Fletcher‐Watson, S. (2022) Charting the impact of autism and bilingualism for autistic and non‐autistic children, 2018—2020 [Data collection]. Colchester, Essex: UK Data Service. 10.5255/UKDA-SN-855431

[jlcd12939-bib-0009] Davis, R. , Montgomery, L. , Rabagliati, H. , Sorace, A. & Fletcher‐Watson, S. (2021) Charting the impact of bilingualism on social attentional preferences in children with and without autism. Preprint: 10.17605/OSF.IO/75PHW.

[jlcd12939-bib-0010] Drysdale, H. , van der Meer, L. & Kagohara, D. (2015) Children with autism spectrum disorder from bilingual families: a systematic review. Review Journal of Autism and Developmental Disorders, 2(1), 26–38.

[jlcd12939-bib-0055] Edwards, V. (2011). Globalization and multilingualism: The case of the UK. Intercultural Communication Studies, 20(1), 27–35.

[jlcd12939-bib-0011] Gonzalez‐Barrero, A.M. & Nadig, A.S. (2019) Can bilingualism mitigate set‐shifting difficulties in children with autism spectrum disorders? Child Development, 90(4), 1043–1060. 29111575 10.1111/cdev.12979

[jlcd12939-bib-0012] Greenwood, N. , Wright, J.A. & Bithell, C. (2006) Perceptions of speech and language therapy amongst UK school and college students: implications for recruitment. International Journal of Language & Communication Disorders, 41(1), 83–94. 16272004 10.1080/13682820500177604

[jlcd12939-bib-0013] Gunnerud, H.L. , Ten Braak, D. , Reikerås, E.K.L. , Donolato, E. & Melby‐Lervåg, M. (2020) Is bilingualism related to a cognitive advantage in children? A systematic review and meta‐analysis. Psychological Bulletin, 146(12), 1059. 32914991 10.1037/bul0000301

[jlcd12939-bib-0014] Halle, T.G. , Whittaker, J.V. , Zepeda, M. , Rothenberg, L. , Anderson, R. , Daneri, P. , et al. (2014) The social–emotional development of dual language learners: looking back at existing research and moving forward with purpose. Early Childhood Research Quarterly, 29, 734–749. 10.1016/j.ecresq.2013.12.002.

[jlcd12939-bib-0015] Hampton, S. , Rabagliati, H. , Sorace, A. & Fletcher‐Watson, S. (2017) Autism and bilingualism: a qualitative interview study of parents’ perspectives and experiences. Journal of Speech, Language, and Hearing Research, 60, 435–446. 10.1044/2016_JSLHR-L-15-0348 28196376

[jlcd12939-bib-0016] Han, W.J. & Huang, C.C. (2010) The forgotten treasure: bilingualism and Asian children's emotional and behavioral health. American Journal of Public Health, 100(5), 831–838. 20299654 10.2105/AJPH.2009.174219 PMC2853634

[jlcd12939-bib-0017] Harris, B. , Barton, E.E. & Albert, C. (2014) Evaluating autism diagnostic and screening tools for cultural and linguistic responsiveness. Journal of Autism and Developmental Disorders, 44, 1275–1287. 10.1007/s10803-013-1991-8 24186120

[jlcd12939-bib-0018] Howard, K. , Gibson, J. & Katsos, N. (2021a) Parental perceptions and decisions regarding maintaining bilingualism in autism. Journal of Autism and Developmental Disorders, 51, 179–192. 32388758 10.1007/s10803-020-04528-x PMC7810638

[jlcd12939-bib-0019] Howard, K.B. , Katsos, N. & Gibson, J.L. (2021b) Practitioners' perspectives and experiences of supporting bilingual pupils on the autism spectrum in two linguistically different educational settings. British Educational Research Journal, 47, 427–449. 10.1002/berj.3662

[jlcd12939-bib-0059] Howard, K. B. , Katsos, N. , & Gibson, J. L. (2019). The school experiences of bilingual children on the autism spectrum: An interpretative phenomenological analysis. Research in developmental disabilities, 87, 9–20. 30703680 10.1016/j.ridd.2019.01.008

[jlcd12939-bib-0020] Hussein, A.M. , Pellicano, E. & Crane, L. (2019) Understanding and awareness of autism among Somali parents living in the United Kingdom. Autism, 23, 1408–1418. 10.1177/1362361318813996 30486651

[jlcd12939-bib-0056] Jegatheesan, B. (2011). Multilingual development in children with autism: Perspectives of South Asian Muslim immigrant parents on raising a child with a communicative disorder in multilingual contexts. Bilingual Research Journal, 34(2), 185–200.

[jlcd12939-bib-0021] Kandeh, M.S. , Kandeh, M.K. , Martin, N. & Krupa, J. (2020) Autism in black, Asian and minority ethnic communities: a report on the first autism voice UK symposium. Advances in Autism, 6, 165–175. 10.1108/AIA-12-2018-0051

[jlcd12939-bib-0022] Kallio, H. , Pietilä, A.M. , Johnson, M. & Kangasniemi, M. (2016) Systematic methodological review: developing a framework for a qualitative semi‐structured interview guide. Journal of Advanced Nursing, 72(12), 2954–2965. 27221824 10.1111/jan.13031

[jlcd12939-bib-0023] Kay‐Raining Bird, E. , Lamond, E. & Holden, J. (2012) Survey of bilingualism in autism spectrum disorders. International Journal of Language & Communication, 47, 52–64. 10.1111/j.1460-6984.2011.00071.x 22268901

[jlcd12939-bib-0024] Kim, H.U. & Roberti, M. (2014) Tengo que habla español. Yo no entiendo ingles!: a qualitative case study on a bilingual child with Autism Spectrum. Journal of Special Education Apprenticesh, 3, 7.

[jlcd12939-bib-0025] Kinnear, S.H. , Link, B.G. , Ballan, M.S. & Fischbach, R.L. (2016) Understanding the experience of stigma for parents of children with autism spectrum disorder and the role stigma plays in families’ lives. Journal of Autism and Developmental Disorders, 46, 942–953. 10.1007/s10803-015-2637-9 26659549

[jlcd12939-bib-0026] Kovács, Á.M. (2012) Early bilingualism and theory of mind: bilinguals’ advantage in dealing with conflicting mental representations. Access to Language and Cognitive Development, 192–218.

[jlcd12939-bib-0027] Lehtonen, M. , Soveri, A. , Laine, A. , Järvenpää, J. , De Bruin, A. & Antfolk, J. (2018) Is bilingualism associated with enhanced executive functioning in adults? A meta‐analytic review. Psychological Bulletin, 144(4), 394. 29494195 10.1037/bul0000142

[jlcd12939-bib-0028] Kenny, L. , Hattersley, C. , Molins, B. , Buckley, C. , Povey, C. & Pellicano, E. (2016) Which terms should be used to describe autism? Perspectives from the UK autism community. Autism, 20(4), 442–462. 26134030 10.1177/1362361315588200

[jlcd12939-bib-0029] Kremer‐Sadlik, T. (2005) To be or not to be bilingual: autistic children from multilingual families. In Proceedings of the 4th International Symposium on Bilingualism . pp. 1225–1234.

[jlcd12939-bib-0030] Lund, E.M. , Kohlmeier, T.L. & Durán, L.K. (2017) Comparative language development in bilingual and monolingual children with autism spectrum disorder: a systematic review. Journal of Early Intervention, 39(2), 106–124.

[jlcd12939-bib-0031] Marian, V. & Shook, A. (2012) The cognitive benefits of being bilingual. In *Cerebrum: the Dana forum on brain science* (Vol. 2012). Dana Foundation. PMC3583091 23447799

[jlcd12939-bib-0032] Maxwell, J.A. (2012) A realist approach for qualitative research. Thousand Oaks, CA: Sage.

[jlcd12939-bib-0033] Marinova‐Todd, S.H. , Colozzo, P. , Mirenda, P. , Stahl, H. , Bird, E.K.R. , Parkington, K. , … & Genesee, F. (2016) Professional practices and opinions about services available to bilingual children with developmental disabilities: an international study. Journal of Communication Disorders, 63, 47–62. 27814797 10.1016/j.jcomdis.2016.05.004

[jlcd12939-bib-0034] Mdlalo, T. , Joubert, R.W. & Flack, P.S. (2019) The cat on a hot tin roof? critical considerations in multilingual language assessments. The South African Journal of Communication Disorders, 66, 1–7. 10.4102/sajcd.v66i1.610 PMC6556923 31170786

[jlcd12939-bib-0035] Montgomery, L. , Chondrogianni, V. , Fletcher‐Watson, S. , Rabagliati, H. , Sorace, A. & Davis, R. (2022) Measuring the impact of bilingualism on executive functioning via inhibitory control abilities in autistic children. Journal of Autism and Developmental Disorders, 52(8), 3560–3573. 10.1007/s10803-021-05234-y 34406588 PMC9296418

[jlcd12939-bib-0036] Munroe, K. , Hammond, L. & Cole, S. (2016) The experiences of African immigrant mothers living in the United Kingdom with a child diagnosed with an autism spectrum disorder: an interpretive phenomenological analysis. Disability & Society, 31, 798–819. 10.1080/09687599.2016.1200015

[jlcd12939-bib-0037] Opitz, B. & Degner, J. (2012) Emotionality in a second language: it's a matter of time. Neuropsychologia, 50, 1961–1967. 10.1016/j.neuropsychologia.2012.04.021 22569217

[jlcd12939-bib-0038] Panayiotou, A. (2004) Switching codes, switching code: bilinguals’ emotional responses in English and Greek. Journal of Multilingual and Multicultural Development, 25(2–3), 124–139.

[jlcd12939-bib-0039] Papoudi, D. , Jørgensen, C.R. , Guldberg, K. & Meadan, H. (2021) Perceptions, experiences, and needs of parents of culturally and linguistically diverse children with autism: a scoping review. Review Journal of Autism and Developmental Disorders, 8, 195–212. 10.1007/s40489-020-00210-1

[jlcd12939-bib-0040] Pavlenko, A. (2005) Emotions and multilingualism. Cambridge, UK: Cambridge University Press.

[jlcd12939-bib-0041] Pesco, D. , MacLeod, A.A. , Bird, E.K.R. , Cleave, P. , Trudeau, N. , de Valenzuela, J.S. , … & Verhoeven, L. (2016) A multi‐site review of policies affecting opportunities for children with developmental disabilities to become bilingual. Journal of Communication Disorders, 63, 15–31. 27814795 10.1016/j.jcomdis.2016.05.008

[jlcd12939-bib-0042] Pert, S. & Bradley, B. (2018) Clinical guidelines for speech and language therapists: Bilingualism: Working with bilingual clients/patients with speech, language and communication needs.

[jlcd12939-bib-0043] Selman, L.E. , Fox, F. , Aabe, N. , Turner, K. , Rai, D. & Redwood, S. (2018) ‘You are labelled by your children's disability’ – a community‐based, participatory study of stigma among Somali parents of children with autism living in the United Kingdom. Ethnicity & Health, 23, 781–796. 10.1080/13557858.2017.1294663 28277014

[jlcd12939-bib-0044] Sharaan, S. , Fletcher‐Watson, S. & MacPherson, S.E. (2021) The impact of bilingualism on the executive functions of autistic children: a study of English–Arabic children. Autism Research, 14(3), 533–544. 33241665 10.1002/aur.2439

[jlcd12939-bib-0045] Sheridan, J. (1999) A career in speech and language therapy: for white women only. Bulletin of the Royal College of Speech & Language Therapists, February, 9.

[jlcd12939-bib-0046] Sher, D.A. , Gibson, J.L. & Browne, W.V. (2022) It's like stealing what should be theirs. An exploration of the experiences and perspectives of parents and educational practitioners on Hebrew–English bilingualism for jewish autistic children. Journal of Autism and Developmental Disorders, 52(10), 4440–4473. 34655375 10.1007/s10803-021-05314-z PMC8520336

[jlcd12939-bib-0047] Szczepura, A. (2005) Access to health care for ethnic minority populations. Postgraduate Medical Journal, 81, 141–147. 10.1136/pgmj.2004.026237 15749788 PMC1743229

[jlcd12939-bib-0048] Uljarević, M. , Katsos, N. , Hudry, K. & Gibson, J.L. (2016) Practitioner review: multilingualism and neurodevelopmental disorders – an overview of recent research and discussion of clinical implications. Journal of Child Psychology and Psychiatry, 57, 1205–1217. 10.1111/jcpp.12596 27443172

[jlcd12939-bib-0049] Valicenti‐McDermott, M. , Tarshis, N. , Schouls, M. , Galdston, M. , Hottinger, K. , Seijo, R. , et al. (2013) Language differences between monolingual English and bilingual English–Spanish young children with autism spectrum disorders. Journal of Child Neurology, 28, 945–948. 10.1177/0883073812453204 22859698

[jlcd12939-bib-0050] Wang, M. , Jegathesan, T. , Young, E. , Huber, J. & Minhas, R. (2018) Raising children with autism spectrum disorders in monolingual vs bilingual homes: a scoping review. Journal of Developmental & Behavioral Pediatrics, 39(5), 434–446. 29746381 10.1097/DBP.0000000000000574

[jlcd12939-bib-0051] Wilder, L.K. , Dyches, T.T. , Obiakor, F.E. & Algozzine, B. (2004) Multicultural perspectives on teaching students with autism. Focus on Autism and Other Developmental Disabilities, 19(2), 105–113.

[jlcd12939-bib-0052] Yu, B. (2013) Issues in bilingualism and heritage language maintenance: perspectives of minority‐language mothers of children with autism spectrum disorders. American Journal of Speech‐Language Pathology, 22(1), 10–24. 10.1044/1058-0360(2012/10-0078) 23071196

[jlcd12939-bib-0053] Zhang, J. , Wheeler, J.J. & Richey, D. (2006) Cultural validity in assessment instruments for children with autism from a Chinese cultural perspective. International Journal of Special Education, 21, 109–114.

